# Alarming Rise in Global Rabies Cases Calls for Urgent Attention: Current Vaccination Status and Suggested Key Countermeasures

**DOI:** 10.7759/cureus.50424

**Published:** 2023-12-13

**Authors:** Priyabrata Pattnaik, Ahmed Mahal, Snehasish Mishra, Anas Alkhouri, Ranjan K Mohapatra, Venkataramana Kandi

**Affiliations:** 1 Human Biologicals, Indian Immunologicals Ltd., Hyderabad, IND; 2 Department of Medical Biochemical Analysis, College of Health Technology, Cihan University-Erbil, Erbil, IRQ; 3 School of Biotechnology, Kalinga Institute of Industrial Technology (KIIT), Bhubaneswar, IND; 4 College of Pharmacy, Cihan University-Erbil, Erbil, IRQ; 5 Department of Chemistry, Government College of Engineering, Keonjhar, IND; 6 Department of Clinical Microbiology, Prathima Institute of Medical Sciences, Karimnagar, IND

**Keywords:** one-health approach, key countermeasures, vaccination status, dog-mediated rabies, rabies

## Abstract

In the wake of rising rabies cases worldwide, especially after the COVID-19 pandemic, it is time to understand the scenario better and suggest technically sound and plausible countermeasures. This article is an attempt at this perspective. Although a critical zoonotic viral disease, rabies is preventable. Medico-legally, the ailment is classified as furious rabies and paralytic rabies. The four world bodies, namely, the World Health Organisation (WHO), the Food and Agriculture Organisation (FAO), the World Organisation for Animal Health (OIE), and the Global Alliance for Rabies Control (GARC) endorsed framing a global support system to eradicate human death from dog-mediated rabies under the 'Zero by 30' framework. The framework calls for extending the vaccination of dogs to reduce the risk of human rabies. Stray dogs became aggressive primarily due to their food shortage during the pandemic lockdown. As many adopted stray dogs were disowned post-pandemic, decreased human-dog interactions increased the aggressiveness among dogs. As a result, 'dog-bite' cases rose, with a sudden spike in rabies cases and dog-bite-induced deaths in India and elsewhere. Jeopardising the ‘Zero by 30’ plan is certainly a public health concern. Stray dog sterilisation through the irreversible ductal occlusion technique and reversible inhibition of sperm under guidance (RISUG) are other suggested interventions to control rabies. Importantly, wildlife like foxes, raccoons, skunks, and bats could also be rabid. Three out of the four WHO-pre-qualified human vaccines against rabies are intradermally administered as post-exposure prophylaxis, while the intramuscular one is more popular. Even though 'Zero by 30' may not be achieved within the set timeframe, it is time for a concerted and planned strategy by global agencies to curb the globally rising rabies cases and manage the disease better. The 'One Health' model seems to be a plausible guideline and the ultimate countermeasure to achieve this.

## Editorial

Although rabies is a critical zoonotic viral disease, it is preventable. Frequently spreading through the bite of a rabid animal, it causes progressive and fatal brain and spinal cord inflammation. Based on the way it manifests, the ailment is medically classified into two types: furious rabies (characterised by hyperactivity and hallucinations) and paralytic rabies (characterised by paralysis and coma). Globally, rabies is estimated to cause 59,000 human deaths in more than 150 countries, the majority (95%) of which occur in African and Asian countries [1]. Given that rabies is a globally prevalent neglected tropical disease and that many rabies cases remain either unreported or underreported, the extent of the disease remains blurred. Dog-mediated rabies is allegedly eliminated from regions like the Americas, Australia, Canada, Western Europe, and a few other countries in the Pacific. Although India accounts for more than 35% of the globally reported dog-mediated rabies deaths, an analysis of the WHO’s data with the Global Health Observatory (GHO) indicates that many countries have failed to provide credible data on the annual deaths. In December 2015, the World Health Organisation (WHO), the Food and Agriculture Organisation (FAO), the World Organisation for Animal Health (OIE), and the Global Alliance for Rabies Control (GARC) collectively endorsed a global support system to eradicate human death from dog-mediated rabies by 2030 [2]. The 'Zero by 30' framework is a global strategy to implement pragmatic changes in approximately 100 countries over the decade. Among other activities, the plan calls for extending dog vaccine usage to reduce the risk of human rabies and increase access to human vaccines, particularly for the high-risk population [2]. As the majority of the cases were associated with dog-transmitted viruses involving the Asian and African population, the 'Zero by 30' framework was devised to increase public awareness, dog vaccination, and vaccinating a human pre- and post-exposure as prophylaxis to ultimately achieve zero death due to rabies by 2030. The 'Zero by 30' framework also advocates a unified surveillance mechanism and collaborative ambience between human and animal healthcare, allowing for better financial and resource management by participating countries under the framework.

The COVID-19 pandemic affected the global human population in general by limiting access to healthcare facilities due to overcrowding, because of which managing regular illnesses and dog-bite cases could not be effectively ensured. The lockdowns, restricted public movements, and general transport due to the COVID-19 pandemic hindered the availability of pre- and post-exposure prophylactic measures. The pandemic also affected the implementation of mass vaccinations for dogs. Other factors that contributed to the difficulties in rabies management during the COVID-19 pandemic included increased aggressiveness in dogs, an altered dog population, risky human-dog encounters, and disrupted surveillance systems. Global adoption of stray dogs increased during the recent COVID-19 pandemic, which contributed to reduced dog-bite cases [3]. Pets during the pandemic seemingly played an essential role in mitigating loneliness and facilitating the mental health of the owner [4,5]. However, the COVID-19-pandemic-induced lockdown led to mismanagement of the handling of stray dogs, their vaccination, and birth control activities [6]. Stray dogs developed aggressive behaviour due to the shortage of food during the lockdown. Post-peak COVID-19, offices opened and the public started moving out for work, whereby social distancing vanished gradually as the pandemic lockdown withdrew, and as a result, many adopted dogs were disowned. The post-COVID abandonment of pets and a decreased human-dog interaction resulted in increased aggressive behaviour among dogs and a proportional increase in dog bites [7]. There was a sudden spike in rabies cases and dog-bite-induced deaths in India and many other countries after the COVID-19 pandemic. Rising cases were reported by the National Institute for Communicable Diseases (NICD) as compared to earlier years [8]. Rabies cases in Maharashtra, India, doubled during the initial phase of the pandemic. Although treated with immunoglobulin and a vaccine against rabies, a 12-year-old child died in Kerala, India, attributed to rabies caused by a dog bite in the head that rapidly infected the central nervous system. Usually, dog-bite cases rise during the summer, especially in school-age children. While the lower extremity is usually bitten by teenagers, toddlers disproportionately suffer injuries to the head, obviously due to their short height [9]. The Department of Zoonotic Diseases Centre for Communicable Diseases Control and Prevention (Iran) reported recently that deaths by dog-bite-induced rabies increased roughly three times in 2021 (16 deaths) in comparison to six deaths in 2018. It was also observed that the number of dog bites in the COVID-19 era doubled in comparison to pre-pandemic times. Africa has reported the largest number of rabies cases after Asia, where the annual dog-mediated rabies death rate is about 21,500. There have been recent rabies outbreaks in Kenya, South Africa, Ghana, Zimbabwe, Morocco, and elsewhere in Africa. It is certainly a public concern, and unless concerted and organised efforts are in place, the ‘Zero by 30’ plan may be jeopardised.

Human rabies is preventable through prompt and appropriate medical care. The US reports dog-mediated rabies as rare. Annually, about 60,000 Americans who are bitten or scratched by a suspected (potentially infected) animal get post-exposure prophylaxis (PEP) every year. An attack by wild animals was reported recently. In a rare human rabies case, a bat-bitten child died in Texas, USA, as no immediate treatment was sought due to a non-discernible bite mark. The Centres for Disease Control and Prevention (CDC) has initiated awareness campaigns about the risks of bats in the USA after three people died of rabies between late September and early November 2021 [10]. More than 90% of the reported rabies cases in the USA are due to animals in the wild; wildlife like foxes, raccoons, skunks, and bats could be rabid. Contacts with infected bats are the leading cause of human rabies deaths in this country. Even an incomprehensively small bat scratch or bite can still spread rabies. Most pets get rabies from having contact with wildlife. Pets (cats and dogs) and livestock (cattle and horses) can also be rabid. Nearly all the pets and livestock that get rabies were observed to have not been vaccinated or were not vaccine-updated. Rabies due to stray dogs is common in India [11]. The rabies virus travels to the brain before it manifests the inflammation symptoms, which may begin weeks or months after exposure. As pets can get rabies from rabid wildlife and can transmit it to humans, preventing rabies in them is a key to avoiding human rabies.

Human rabies vaccines are recommended as pre-exposure prophylaxis for someone at high risk of exposure to the rabies virus and post-exposure too, which accounts for 97% of global rabies vaccine use. The rabies vaccine manufacturer base is extremely fragmented, with 24 manufacturers in all. Most (85%) of the rabies vaccine supply is from manufacturers in China and India. There are only four WHO-pre-qualified vaccine products for supply. Three of them are labelled for intradermal (ID) administration and available in a 1.0 mL vial presentation. The supply seems to be enough to cater to the usual global requirement [12]. However, owing to the supply-chain breakdown during the pandemic, the rabies vaccine shortage affected several countries in 2019; some also had challenges in procuring the vaccines.

Most countries (181 countries by estimate) recommend intramuscular (IM) rabies vaccine administration for PEP. Six countries, namely, Côte d’Ivoire, Cambodia, India, Nepal, Pakistan, and Vietnam, use a combined IM and ID regimen, and an additional six countries (Bangladesh, Bhutan, Madagascar, the Philippines, Sri Lanka, and Thailand) use only the WHO-recommended Institut Pasteur Cambodge (IPC) ID regimen. Mostly China-driven, WHO's global market report indicates that a switch to ID administration is likely to reduce annual vaccine needs considerably from an estimated 60 million vials to about 20 million [12]. The COVID-19 pandemic had an unfamiliar global impact on the demand for rabies vaccine vials. Surveys to establish realistic figures on global vaccine demand are of top priority. PEP is an effective emergency response to any rabies exposure, preventing the virus from entering the central nervous system. The immediate post-exposure responses include (1) washing the wound externally with water and soap for at least 15 minutes after a suspected exposure, (2) administering a potent and effective WHO-standardised rabies vaccine course, and (3) administering rabies immunoglobulin and/or monoclonal antibodies.

One of the other proposed interventions other than vaccines to control rabies is reducing the number of stray dogs through contraception. For this, the irreversible ductal occlusion technique injects sclerosing chemicals into the lumen of the ductus percutaneously in dogs with excipients like ethanol, silver nitrate, acetic acid, and formaldehyde [13]. Reversible inhibition of sperm under guidance (RISUG), which has been successfully tried in humans and langurs, is a male non-surgical reversible contraceptive technique to control reproduction that could be tried in dogs and cats to inhibit sperm formation [14]. However, the efficacy of the technique on stray dogs needs to be validated. RISUG involves adding (or without the addition of) dimethylsulfoxide to styrene maleic anhydride and injecting it into the ductus deferens. With a dual effect, it physically blocks the lumen of the ductus deferens and chemically reduces the pH locally [15]. The latter (RISUG) process is relatively cumbersome and could often be reverted. There are certain limitations and challenges, too. These include, but are not limited to, those detailed below. Logistics limitations include physically catching a stray dog to sterilise it and deploying the mechanism at the field level. The economics of field implementation of RISUG in resource-limited developing countries are yet to be ascertained. Similarly, the technical issue is that large-scale cGMP-grade manufacturing of the active compound of RISUG is yet to be established.

As observed, dogs are the predominant source of rabies in developing countries, while wild animals are the primary carriers of rabies in developed countries. Canine rabies contributes to 99% of human deaths annually and is more critical, especially in the third world, from a public health perspective. As rabies in isolated countries like Australia and Western Europe is not reported, they are regarded as rabies-free. Rabies cases have reportedly declined in Bangladesh, the Philippines, Sri Lanka, Tanzania, Vietnam, and South Africa. The prevalence of rabies in India is due to low awareness about pre- and post-exposure prophylactic measures, a non-restricted canine population, and poor dog vaccination. In order to help address this disease, Indian Immunologicals Ltd. (IIL) successfully runs the ‘Fearless Against Rabies’ campaign [16]. The digital programme intends to make the public and medical professionals aware of rabies-related aspects. The post-pandemic recovery plan needs the government, policy, administration, and logistics to also augment human rabies prophylaxis and vaccination of stray dogs and wild animals against rabies [17]. Mass vaccination of canines against rabies could be accelerated to decrease the risk related to the bite of stray and pet dogs in human habitats, especially in the backdrop of the recurrent global wave of the COVID-19 pandemic, which is looming large to occur sooner than later [18]. The ‘dog-mediated human rabies elimination programme’ is a novel One Health approach to meaningfully engaging animal healthcare to achieve zero zoonotic diseases by the year 2030. The human and animal health departments may collectively work in concerted efforts to achieve the objective. Critical strategies to control and prevent rabies could include (1) raising awareness about it, (2) administering PEP, (3) using rabies immunoglobulins, (4) dog mass vaccination, and (5) vaccinating other pets and livestock wherever possible.

The 'One Health' model is a low-cost but effective strategy to reduce rabies-associated death and possibly eliminate rabies. Following the WHO’s commitment to true practice is vital, which may include increased disease surveillance and raising awareness, particularly among the deprived community. The National Bridging Workshop on Rabies (NBW-R) to support the implementation of effective rabies control programmes was introduced. The workshop basically focused on forging collaborations across various sectors at the animal-human-environment interface to control rabies following the 'One Health' principles. The workshop in Ghana on 15-17 August 2022 was followed by a second workshop in Bali on 12-14 April 2023. Conducting such programmes is encouraging and necessary to achieve the global goal of 'Zero by 30'. As India reported about one-third of the global burden of rabies, it can actively participate in it to play a key role in achieving 'Zero by 30'. Rabies is transmitted by numerous animals (including the ones in the wild) and not only dogs. Keeping this in view, a protracted suggestion could be vaccine development and a mass vaccination drive in these animals as prophylaxis can be ensured. Wild animals coming inadvertently into contact with humans may be identified and, if possible, vaccinated either as a food additive or as an epidermal application. These supposedly vaccinated animals may go back to their habitat in the wild and disseminate herd immunity that would ensure herd vaccination.

Conclusion

Considering the grim and alarming scenario of the rising rabies cases, especially in the post-COVID-19 pandemic world, there is an urgent need to address it with a 360° approach to achieve the targeted 'Zero by 30' as endorsed as a global support system by the WHO, the FAO, the OIE, and the GARC to eradicate rabies-mediated human deaths. Being a zoonotic viral disease, vaccination drives for various domesticated/pet and wild carrier animals, including dogs, need to be considered besides vaccinating humans. Further, steps to segregate the wildlife and restrict them from the human-habitat interface need to be taken so that the viral transmission risk is reduced. Along with the same, measures need to be in place for seamless vaccine supply across the globe and should be available to the needy population on short notice. Making the public aware of the basic individual-level precautionary and corrective steps is also necessary. Beyond the vaccination intervention, another plausible intervention seems to be the contraception of the carrier animals, including dogs. Mass vaccination campaigns covering up to 70% of the dog population are suggested to manage the dog population and thus break the chain. With all these various required steps in view for a 360° solution to the issue, a concerted effort by healthcare agencies and health professionals (that include doctors, veterinarians, and sectoral researchers) to forge a global nexus in line with the 'One Health' model seems plausible and tactically useful. Awareness through conferences and seminars in the NBW-R lines as well as the 'Fearless Against Rabies' campaign by the IIL are encouraging initiatives to develop public awareness and public participation. Increasing rabies cases primarily through dog bites in African and Asian countries need thorough surveillance and effective countermeasures in line with the 'One Health' approach.
